# Lagovirus europeus GI.2 (rabbit hemorrhagic disease virus 2) infection in captive mountain hares (*Lepus timidus*) in Germany

**DOI:** 10.1186/s12917-020-02386-4

**Published:** 2020-05-27

**Authors:** Melanie Buehler, Sonja T. Jesse, Heike Kueck, Bastian Lange, Patricia Koenig, Wendy K. Jo, Albert Osterhaus, Andreas Beineke

**Affiliations:** 1 Institute of Pathology, University of Veterinary Medicine Hanover, Foundation, Buenteweg, 17 30559 Hannover, Germany; 2 Research Center for Emerging Infections and Zoonoses, University of Veterinary Medicine Hanover, Foundation, Buenteweg, 17 30559 Hannover, Germany; 3 Zoo am Meer Bremerhaven, H.-H.-Meier-Straße 7, 27568 Bremerhaven, Germany; 4 Institute of Diagnostic Virology, Friedrich-Loeffler-Institut, Federal Research Institute for Animal Health, Suedufer 10, 17493 Greifswald, Insel Riems Germany

**Keywords:** Leporidae, Caliciviridae, Emerging infection, Phylogenetic analysis, Rabbit hemorrhagic disease virus 2, RHDV2, Lagovirus, GI.2

## Abstract

**Background:**

Rabbit hemorrhagic disease virus (RHDV, Lagovirus europeus GI.1) induces a contagious and highly lethal hemorrhagic disease in rabbits. In 2010 a new genotype of lagovirus (GI.2), emerged in Europe, infecting wild and domestic population of rabbits and hares.

**Case presentation:**

We describe the infection with a GI.2 strain, “Bremerhaven-17”, in captive mountain hares (*Lepus timidus*) in a zoo facility in Germany. *Postmortem* examination revealed RHD-like lesions including necrotizing hepatitis. RT-qPCR and AG-ELISA confirmed presence of GI.2. Recombination and phylogenetic analysis grouped the identified strain with other GI.2 strains, sharing nucleotide identity of 91–99%.

**Conclusion:**

Our findings confirm that mountain hares are susceptible to GI.2 infection, due to a past recombination event facilitating virus spillover from sympatric rabbits*.*

## Background

*Rabbit hemorrhagic disease virus* (RHDV) is a hitherto species-specific lagovirus of the *Caliciviridae* family that has a single stranded, positive-sense RNA genome. RHDV has been circulating in Europe since the 1980s and infects wild and domestic rabbits (*Oryctolagus cuniculus)* generally older than 2 months. The virus binds to histo-blood group antigens (HBGA) and shows a tropism to hepatocytes, macrophages and endothelial cells [1–3]. Suspected viral entry sites are the tracheal and duodenal mucosa [1–4] Aside from hemorrhages in several organs, affected rabbits show pathognomonic necrosis of hepatocytes and hyaline thrombi in renal corpuscles [4]. In 2010, a new genotype of lagoviruses, GI.2 (also known as RHDV2), with a diverse VP60 capsid protein emerged and replaced previously circulating RHDV (GI.1) strains [5–7]. This new variant causes lethal infections also in juvenile (< 2 month old) and RHDV vaccinated rabbits [6]. After its first detection in France, GI.2 spread rapidly and infections were reported in several European countries, North America, the Middle East, and Australia [8].

The *European brown hare syndrome virus* (EBHSV, genotype GII.1), a genetically closely related lagovirus, causes similar lesions and mortality rates as RHDV but exclusively affects hares [9, 10]. Lytic necrosis of liver lobules, fatty degeneration and congestion, as well as hemorrhages are more frequently seen in EBHSV than in RHDV infections, but discriminating between the two entities solely on morphological criteria is difficult [11]. In addition to the European brown hare (*Lepus (L.) europeus*), EBHSV infections have been reported also in other members of the family *Leporidae*, such as mountain hares (*L. timidus)* [12]*,* Italian hares (*L. corsicanus*) [13]*,* Cape hares (*L. capensis*) [14] and eastern cottontail rabbits (*Sylvilagus floridanus*) [15]. Experimental GI.1 infections of European brown hares induces virus-specific antibodies, but fails to cause clinical symptoms in this species [9]. In a retrospective study, GI.1 was found in Iberian hares (*L. granatensis*) in Portugal [16]. In more recent years, GI.2 infections of one Italian hare (*L. corsicanus*) [17], seven Cape hares (*L. capensis mediterraneus*) [15] and European hares (*L. europaeus*) [18] have been reported in different European countries. In addition, GI.2 has been detected in European hares in Australia [19]. Infected hares were found in regions where rabbits also succumbed to GI.2 infection, suggesting viral transmission from rabbits to hares [17, 18, 20]. Recently, RHDV2 infections have been reported in mountain hares from a Swedish island [21]. Different susceptibilities of wild hare populations to GI.2 infection have been observed, most likely due to epidemiological factors such as the density of sympatric rabbits, and genetic factors such as host glycan expression for viral attachment [3, 17]. Moreover, concurrent parasitic infections or a poor nutritional state both suggest a predisposition for fatal GI.2 infections in mountain hares in Sweden [21]. The present report describes pathological findings, recombination and phylogenetic analysis of a GI.2 strain in mountain hares from an outbreak in a zoo facility in Germany.

## Case presentation

**Fig. 1:**
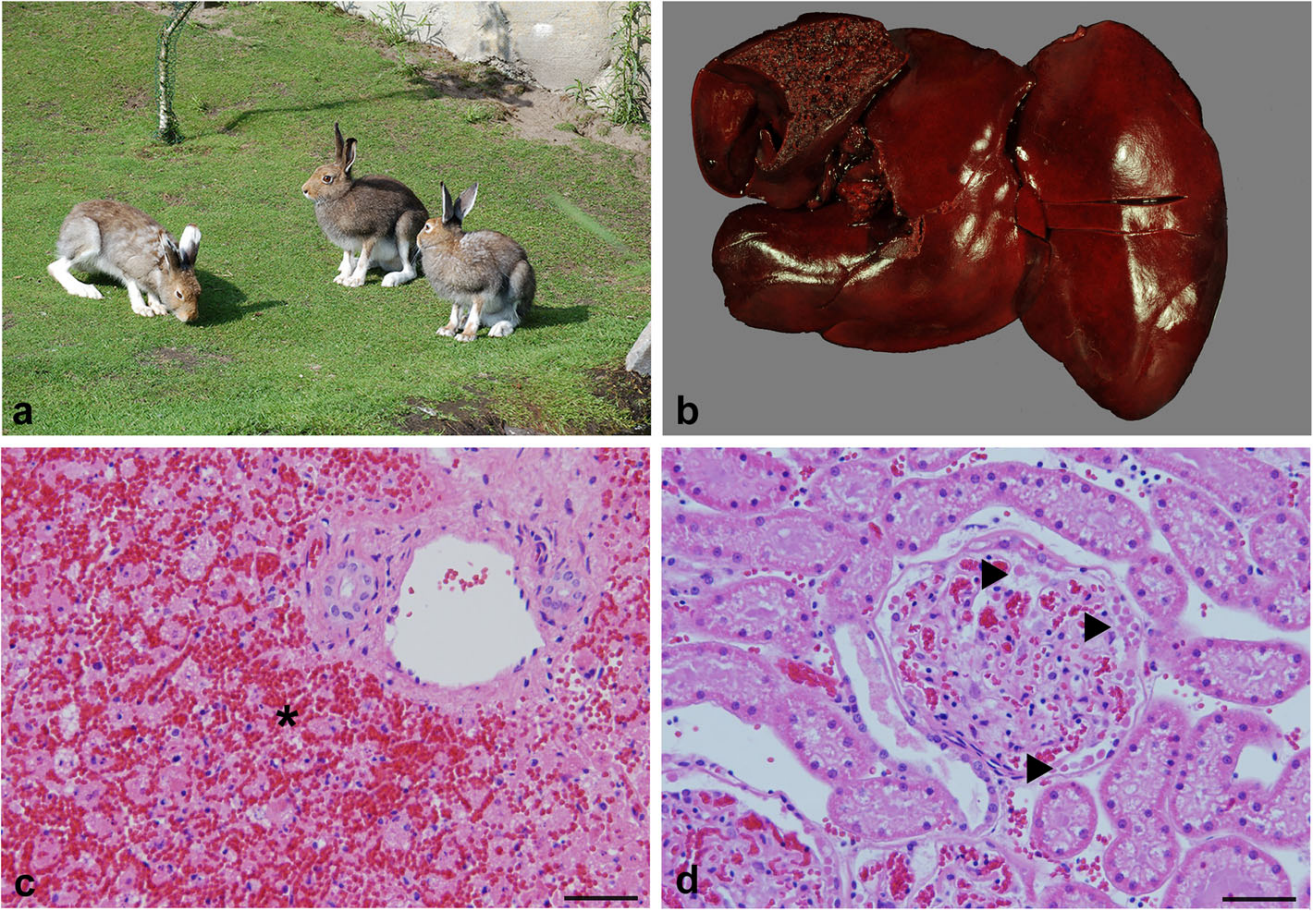
GI.2 infection in captive mountain hares (*Lepus timidus*). **a** Mountain hares in summer coat in the zoo facility in Germany. **b** Macroscopic picture of an infected liver. The liver of GI.2 infected mountain hares presented with friable consistency and moderate to severe congestion. **c** Microscopic lesion within the liver included severe, diffuse, panlobular, necrotizing hepatitis with massive congestion and acute hemorrhages (*). **d** Hyaline thrombi (►) are present within glomerular capillaries

In May 2017, five out of six mountain hares (Supplementary material 1) of a zoo facility in northern Germany (Fig. 1a) died acutely within 2 weeks. The hares (#2, 3, 4) were not vaccinated against GI.2. The two remaining hares (#5, 6) were vaccinated (ERAVAC, Hipra, Amer, Spain) shortly after receiving the necropsy results from the first three hares, but #5 also died soon after. Necropsy of the two adult females (#2, 5) one juvenile female (#3) and male (#4) hares revealed a good nutritional state. The liver of all hares showed a friable consistency and a moderate to severe congestion (Fig. 1b). Further findings included diffuse congestions of lungs, kidneys and spleens. Histologic examination showed severe, panlobular necrotizing hepatitis with massive congestion and acute hemorrhages in all investigated mountain hares (Fig. 1c). The kidneys exhibited several hyaline thrombi within glomerular tufts (Fig. 1d). Viral infection was confirmed in all four examined mountain hares by RT-qPCR (forward primer: 5′–ACT TGT CAG AAC TTG TTG ACA-3′, reverse primer: 5′-TCA GAC ATA AGA AAA GCC ATT AG-3′, probe: 5′-FAM-CCA CAA GCA CGC TTG TGT ACA ACT TG-BHQ1–3′) [22] and commercial antigen ELISA (Eurofins Ingenasa, Madrid, Spain) (Supplementary Material 1) of frozen liver samples, which was performed as previously described [23]. Genome was obtained by next generation sequencing (NGS) to further classify the GI.2 strain [24]. Briefly, liver tissue of animal #5 was processed as previously described [25]. DNA library was prepared according to manufacturer’s protocol (Nextera XT DNA Library Preparation Kit; Illumina) and sequenced on an Illumina NextSeq 500 sequencer with the NextSeq 500/550 High Output Kit v2 (2 × 150 bp paired-end; Illumina). Quality and adapter trimming, as well as reference assembly was performed using CLC Genomics Workbench (v12). The newly discovered strain was named “Bremerhaven-17” (accession number MN901451).

**Fig. 2:**
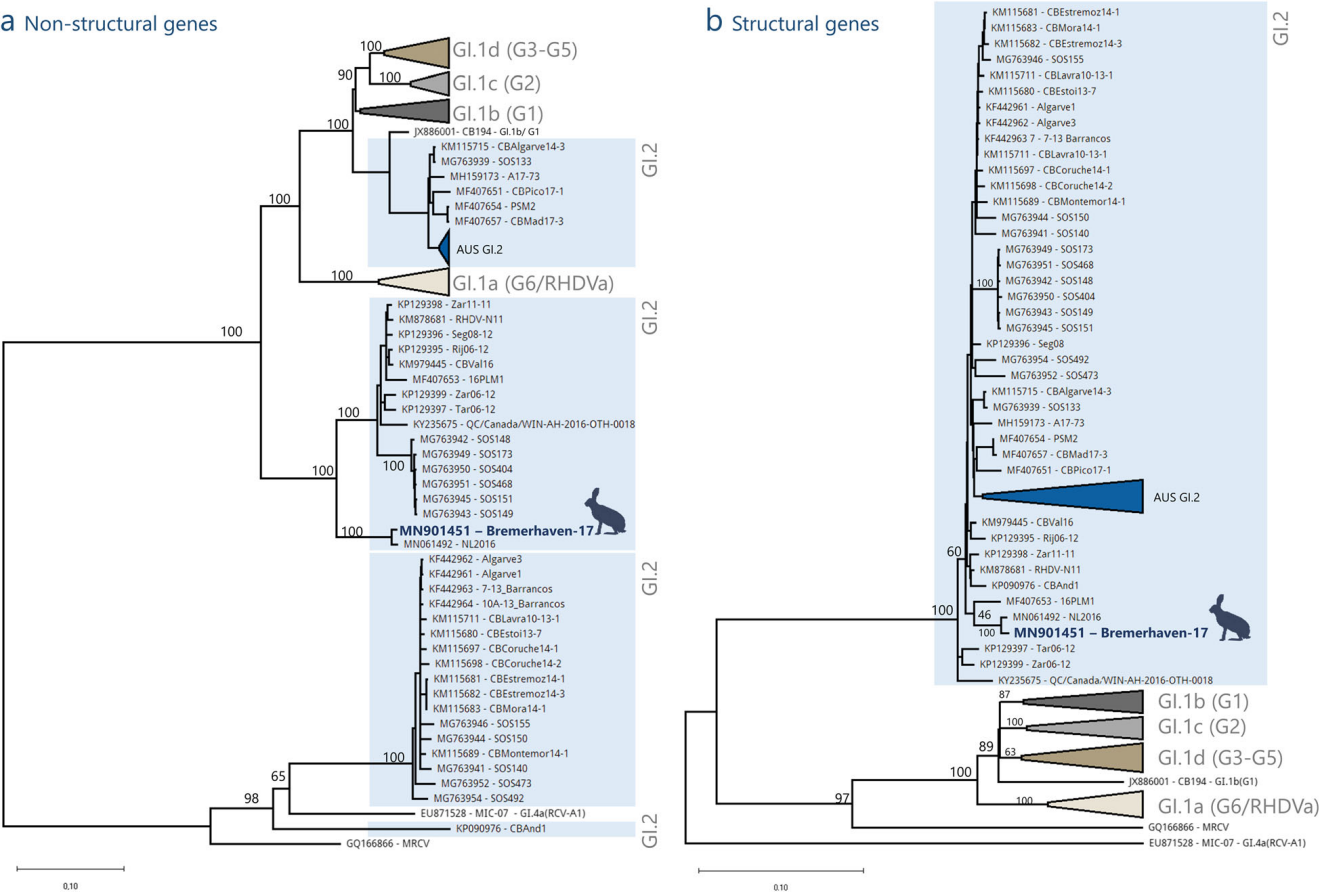
Phylogenetic analysis of the non-structural and structural genes in GI lagoviruses. Two phylogenetic trees were constructed using a representative subset of published lagoviruses (*n* = 85) Phylogenic tree of non-structural genes (**a**) depicts Maximum likelihood phylogenies before the calculated recombination break (5242-5345 nt) using nucleotide positions: 1–5294, In phylogeny of structural genes (VP60 and VP10) (**b**) the Maximum likelihood tree was constructed using nucleotide positions: 5295–7375. Accession numbers of all sequence genomes from Genbank, along with their strain names are indicated in the taxon names. Strains, which belong to GI.2, are highlighted in blue. The newly sequenced “Bremerhaven-17” (accession number MN901451) is shown in bold face. The genotypes GI.1a, GI.1b, GI.1c, GI.1d, along with the Australian GI.2 clades (“AUS GI.2”) previously published [19] are collapsed due to their large number. The scale bar is proportional to the number of nucleotide substitutions per site. Depicted are the bootstrap values of the major nodes. All sequences used for the phylogenetic analyses, along with their new and old classification, can be found in the supplementary material 2

For recombination analysis, a set of sequences (*n* = 388) all belonging to the GI genogroups of lagoviruses, defined by Le Pendu and colleagues [26] was used. The complete genome of “Bremerhaven-17”, excluding the 5’and 3′ UTR, was aligned with a set of sequences of lagoviruses of GI with nucleotide lengths over 7.239 bp found on Genbank using the MAFFT tool (version 7) [27]. The obtained dataset was subsequently used for our recombination analysis using the RDP4 software (version 4.1) [28] applying parameters as described previously [29]. Our results correspond with the findings of previous studies [19, 29, 30], showing a distinct recombination breakpoint in the span of 5242-5345 nt shortly before the open reading frame of the structural proteins VP60 and VP10 (data not shown), splitting the genome into non-structural and structural genes (Fig. 2a).

Prior to phylogenetic analyses of the split genome, a subset of lagovirus sequences (*n* = 85) were aligned using MAFFT (version 7) [27]. All 5′ and 3′ UTRs were cropped resulting in a uniform nucleotide length of 7375. Two phylogenetic trees representing the non-structural genes (nucleotide position 1–5294, representing sequence before the recombination breakpoint) and structural genes (Fig. 2b) (nucleotide position 5295–7375, representing sequence after the recombination breakpoint) were constructed in the same manner: Phylogenies were built using MEGA X [31] with a GTR + G + I model of nucleotide substitution and a bootstrap support of 1000 replications. The structural and non-structural genes of “Bremerhaven-17” clearly cluster with other European strains of GI.2. The highest homology (99.09%) can be found with the strain, “NL2016”, detected in a wild rabbit from the Netherlands in 2016 (accession number MN061492) [32].

## Discussion and conclusions

The herein described pathological findings along with molecular and antigen detection methods confirm the sensitivity of the captive mountain hares to GI.2, as previously observed in wildlife mountain hares [21]. As observed in other vulnerable lagomorphs, all four examined mountain hares died spontaneously and showed RHD-like pathology with severe diffuse necrotizing hepatitis and circulatory disturbances [17, 18, 20, 21]. In contrast to GI.1, GI.2 is known to infect rabbits younger than 10 weeks of age [22, 33]. Correspondingly, in the present report, two juvenile (4 weeks old) mountain hares have succumbed to the virus infection. Before 2010, lagoviruses were thought to be strictly host specific in leporids, with rabbits susceptible to GI.1 and hares to EBHSV infection [11, 26].

The emergence of GI.2 infections of hares in Italy [17, 18, 20], Spain [18], Australia [19], Sweden [21] and the present case clearly indicate the capability of GI.2 to cross species barriers, at least within the taxonomic family Leporidae. In all reported cases, rabbits were infected with GI.2 in the same habitats, suggesting viral spillover from sympatric rabbits to hares [16–20]. In this case, an infection of GI.2 was previously detected in rabbits housed in proximity to the enclosure of the mountain hares (distance four meters) and might have caused infection in the present outbreak. In contrast to GI.2 infected wild mountain hares found in Sweden [21], these captive mountain hares were in good body condition without preexisting diseases or parasitosis, suggesting that clinical manifestation of GI.2 infection can also occur in immunocompetent mountain hares. However, high infection pressure and viral circulation in the zoo facility might have fostered a spillover of GI.2 in the described scenario.

An annuated way of constructing phylogenetic trees in GI is done by sequencing the VP60 gene coding for a major structural protein: In this case the structural genes of “Bremerhaven-17” cluster with other European GI.2 strains. This phylogeny corresponds with the phylogenetic tree found in Fig. 2b.

However, recent findings [19, 29, 30] have shown the importance of sequencing the non-structural genes to appreciate a better understanding of evolutionary recombination events. The non-structural genes of “Bremerhaven-17” clusters within one of the four groups previously described [34] of GI.2. (Fig. 2a). The non-structural genes of Bremerhaven-17 share a common ancestor with G1.1a as opposed to other GI.2 strains, which share a common ancestor with non-pathogenic lagoviruses (GI.3 and GI.4). These recombination events found in the contemporary GI.2 strains clearly have led to an evolutionary advantage, however the true meaning regarding advantages in viral fitness, pathogenicity, host and tissue tropism rely on further studies.

We therefore advocate, continued monitoring, along with full genome sequencing of lagovirus cases. It is crucial to continuously determine the most common types of recombinants found in lagomorphs to predict the emergence of new recombinants and their impact on rabbit and hare populations. This can only be achieved by further characterizing full lagovirus sequence as opposed to performing phylogenetic trees using the VP60 gene, to ensure a better understanding regarding pathogenicity and epidemiology.

Results confirm that GI.2 causes disease and lethality in mountain hares [21] similar to wild and domestic rabbits and other susceptible lagomorphs [17–20]. Cross-species transmission of GI.2 should be considered in the hygienic management of captive mountain hares, demonstrating the need for vaccination strategies for GI.2, reducing the exposure of mountain hares to rabbits and other GI.2 susceptible hare species in endemic areas. Further monitoring of wild hare populations is required to preserve the endangered species and prevent virus spread.

## Supplementary information

**Additional file 1: Supplementary material 1.** Overview of examined mountains hares from a zoo facility in Germany.

**Additional file2 : Supplementary material 2.** Sequences used for the phylogenetic analyses, along with their new and old classification.

## Data Availability

The RHDV2 strain “Bremerhaven-17” is accessible in Genbank under following number: RHDV 2 strain Bremerhaven-17 MN901451.

## Abbreviations

AG-ELISA: Antigen-Enzyme-linked immunosorbent assay
EBH: European brown hare
EBHSV: European brown hare syndrome virus
L.: Lepus
NGS: Next generation sequencing
RHDV: Rabbit hemorrhagic disease virus
RT-qPCR: Quantitative reverse transcription polymerase chain reaction
