# Supplementary material for: Lagovirus europeus GI.2 (rabbit hemorrhagic disease virus 2) infection in captive mountain hares (Lepus timidus) in Germany
Source: BMC Vet Res. 2020 May 27;16:166. doi: 10.1186/s12917-020-02386-4 (PMC7254734; doi:10.1186/s12917-020-02386-4)
Supplement: Supplementary file 1 — Additional file 1: Supplementary material 1. Overview of examined mountains hares from a zoo facility in Germany. [file 12917_2020_2386_MOESM1_ESM.docx]

|  | **day of death** | **age** | **gender** | **body weight** | **RHDV2 vaccination** | **Liver** | | | **pathohistological lesions** |  |
| --- | --- | --- | --- | --- | --- | --- | --- | --- | --- | --- |
|  |  |  |  |  |  | **RT-qPCR [cq]** | **AG-ELISA [OD]** | **NGS** |  |  |
| #1 | 2017/06/02 | adult | unknown | unknown | unvaccinated | n.i. | n.i. | n.i. | first sudden death; no necropsy |  |
| #2 | 2017/06/05 | 2 years | female | 3.4 kg | unvaccinated | positive (19.59) | positive (0.87) | n.i. | liver, lung, spleen: moderate to severe congestion |  |
|  |  |  |  |  |  |  |  |  | liver: diffuse, severe necrosis |  |
|  |  |  |  |  |  |  |  |  | kidney: single hyaline thrombi within glomeruli |  |
| #3 | 2017/06/03 | 1 month | female | 1.0 kg | unvaccinated | positive (20.16) | positive (0.92) | n.i. | liver: diffuse, severe necrosis |  |
| #4 | 2017/06/02 | 1 month | male | 0.9 kg | unvaccinated | positive (20.04) | positive (0.92) | n.i. | liver: diffuse, severe necrosis |  |
| #5 | 2017/06/08 | 1 year | female | 4.3 kg | 2017/06/07 ERAVAC® | positive (19.10) | positive (0.75) | ✓ | lung: moderate congestion |  |
|  |  |  |  |  |  |  |  |  | liver: diffuse, severe necrosis; |  |
|  |  |  |  |  |  |  |  |  | kidney: single hyaline thrombi within glomeruli |  |
| **Supplementary material 1** Overview of examined Mountains hares from a zoo facility in Germany; n.i.: not investigated | | | | | | | | | | |
