# Supplementary material for: Lagovirus europeus GI.2 (rabbit hemorrhagic disease virus 2) infection in captive mountain hares (Lepus timidus) in Germany
Source: BMC Vet Res. 2020 May 27;16:166. doi: 10.1186/s12917-020-02386-4 (PMC7254734; doi:10.1186/s12917-020-02386-4)
Supplement: Supplementary file 2 — Additional file2 : Supplementary material 2. Sequences used for the phylogenetic analyses, along with their new and old classification. [file 12917_2020_2386_MOESM2_ESM.docx]

| **Supplementary material 2** Sequences used for the phylogenetic analyses, along with their new and old classification | | | |
| --- | --- | --- | --- |
| **Accession Number** | **Strain** | **New Nomenclature** | **Old Nomenclature** |
| AY523410 | CD/China | GI.1a | G6/RHDVa |
| KF677011 | STR2012 | GI.1a | G6/RHDVa |
| KY319031 | BIE | GI.1a | G6/RHDVa |
| KY679905 | STR2014 | GI.1a | G6/RHDVa |
| KY319034 | SKO | GI.1a | G6/RHDVa |
| JF412629 | MB/Canada/WIN-AH-2011-OTH-026 | GI.1a | G6/RHDVa |
| KY235676 | HYD | GI.1a | G6/RHDVa |
| MF598301 | K5_08Q712_BatchRelease1/2008 | GI.1a | G6/RHDVa |
| EF558578 | Eisenhuttenstadt | GI.1b | G1 |
| Z49271 | AST89 | GI.1b | G1 |
| Z29514 | SD | GI.1b | G1 |
| JF438967 | G1 | GI.1b | G1 |
| KP090974 | CB110 | GI.1b | G1 |
| KP090975 | CB154 | GI.1b | G1 |
| JX886002 | CB137_Pt | GI.1b | G1 |
| JX886001 | CB194_Pt | GI.1b | G1 |
| EU003579 | Italy-90 | GI.1c | G2 |
| M67473 | genome | GI.1c | G2 |
| KP144789 | PD | GI.1c | G2 |
| KP144790 | KGM | GI.1c | G2 |
| KT006734 | AUS/SA/FlindersRanges/1999 | GI.1c | G2 |
| U54983 | RHDV-V351 | GI.1c | G2 |
| KU882093 | MAL | GI.1c | G2 |
| EF558576 | Jena | GI.1d | G3-G5 |
| KU882095 | ZD0 | GI.1d | G3-G5 |
| KU882092 | GSK | GI.1d | G3-G5 |
| MH190418 | cun/FR/2000/00-21 | GI.1d | G3-G5 |
| KY622127 | P158 | GI.1d | G3-G5 |
| EF363035 | pJG-RHDV-DD06 | GI.1d | G3-G5 |
| EF558574 | Wika | GI.1d | G3-G5 |
| EF558572 | Frankfurt12 | GI.1d | G3-G5 |
| EF558573 | Frankfurt5 | GI.1d | G3-G5 |
| KM878681 | RHDV-N11 | GI.2 | RHDV2/RHDVb |
| KP129398 | Zar11-11 | GI.2 | RHDV2/RHDVb |
| KP129396 | Seg08-12 | GI.2 | RHDV2/RHDVb |
| KM979445 | CBVal16 | GI.2 | RHDV2/RHDVb |
| KP129395 | Rij06-12 | GI.2 | RHDV2/RHDVb |
| KP129397 | Tar06-12 | GI.2 | RHDV2/RHDVb |
| KP129399 | Zar06-12 | GI.2 | RHDV2/RHDVb |
| MF407653 | 16PLM1 | GI.2 | RHDV2/RHDVb |
| KY235675 | QC/Canada/WIN-AH-2016-OTH-0018 | GI.2 | RHDV2/RHDVb |
| MG763942 | SOS148 | GI.2 | RHDV2/RHDVb |
| MG763943 | SOS149 | GI.2 | RHDV2/RHDVb |
| MG763949 | SOS173 | GI.2 | RHDV2/RHDVb |
| MG763951 | SOS468 | GI.2 | RHDV2/RHDVb |
| MG763950 | SOS404 | GI.2 | RHDV2/RHDVb |
| MG763945 | SOS151 | GI.2 | RHDV2/RHDVb |
| MN061492 | NL2016 | GI.2 | RHDV2/RHDVb |
| MN901451 | Bremerhaven-17 | GI.2 | RHDV2/RHDVb |
| MF421576 | AUS/SA/TIN-2/2016 | GI.2 | RHDV2/RHDVb |
| MF421601 | AUS/SA/COO-1/2016 | GI.2 | RHDV2/RHDVb |
| MF421614 | AUS/SA/WS-1/2016 | GI.2 | RHDV2/RHDVb |
| MF421620 | AUS/SA/COL-2/2016 | GI.2 | RHDV2/RHDVb |
| MF421663 | AUS/SA/NUR-1/2016 | GI.2 | RHDV2/RHDVb |
| MF421662 | AUS/SA/ADL-1/2016 | GI.2 | RHDV2/RHDVb |
| MF421599 | AUS/SA/LYN-1/2016 | GI.2 | RHDV2/RHDVb |
| MF421577 | AUS/SA/WIT-1/2016 | GI.2 | RHDV2/RHDVb |
| MF421665 | AUS/SA/MUN-3/2016 | GI.2 | RHDV2/RHDVb |
| MF421600 | AUS/SA/MUN-1/2016 | GI.2 | RHDV2/RHDVb |
| KM115715 | CBAlgarve14-3 | GI.2 | RHDV2/RHDVb |
| MG763939 | SOS133 | GI.2 | RHDV2/RHDVb |
| MH159173 | A17-73 | GI.2 | RHDV2/RHDVb |
| MF407654 | PSM2 | GI.2 | RHDV2/RHDVb |
| MF407657 | CBMad17-3 | GI.2 | RHDV2/RHDVb |
| MF407651 | CBPico17-1 | GI.2 | RHDV2/RHDVb |
| KF442961 | Algarve1 | GI.2 | RHDV2/RHDVb |
| KF442962 | Algarve3 | GI.2 | RHDV2/RHDVb |
| KF442963 | 7-13_Barrancos | GI.2 | RHDV2/RHDVb |
| KF442964 | 10A-13_Barrancos | GI.2 | RHDV2/RHDVb |
| KM115680 | CBEstoi13-7 | GI.2 | RHDV2/RHDVb |
| KM115697 | CBCoruche14-1 | GI.2 | RHDV2/RHDVb |
| KM115711 | CBLavra10-13-1 | GI.2 | RHDV2/RHDVb |
| KM115689 | CBMontemor14-1 | GI.2 | RHDV2/RHDVb |
| KM115681 | CBEstremoz14-1 | GI.2 | RHDV2/RHDVb |
| KM115683 | CBMora14-1 | GI.2 | RHDV2/RHDVb |
| KM115682 | CBEstremoz14-3 | GI.2 | RHDV2/RHDVb |
| KM115698 | CBCoruche14-2 | GI.2 | RHDV2/RHDVb |
| MG763946 | SOS155 | GI.2 | RHDV2/RHDVb |
| MG763944 | SOS150 | GI.2 | RHDV2/RHDVb |
| MG763941 | SOS140 | GI.2 | RHDV2/RHDVb |
| MG763954 | SOS492 | GI.2 | RHDV2/RHDVb |
| MG763952 | SOS473 | GI.2 | RHDV2/RHDVb |
| KP090976 | CBAnd1 | GI.2 | RHDV2/RHDVb |
| EU871528 | MIC-07 | GI.4a | RCV-A1 (1) |
| GQ166866 | MRCV | GI | MRCV |
